# Three year experience of a clinical cardiovascular genetics program for infants with congenital heart disease

**DOI:** 10.1111/chd.12817

**Published:** 2019-06-21

**Authors:** Gabrielle C. Geddes, Erin Syverson, Michael G. Earing

**Affiliations:** ^1^ Department of Pediatrics Medical College of Wisconsin Milwaukee Wisconsin; ^2^ Herma Heart Institute Children's Hospital of Wisconsin Milwaukee Wisconsin

**Keywords:** cardiovascular genetics, congenital heart disease, genetic testing

## Abstract

**Objective:**

To describe the first 3 years of experience of having an inpatient “cardiogenetics” program which involves medical geneticist assessment of infants with major congenital heart disease (CHD) requiring surgical intervention in the first year of life.

**Patients:**

Patients less than a year of age admitted to Children's Hospital of Wisconsin's Herma Heart Institute for surgical intervention for CHD seen by the cardiogenetics program. Patients with major trisomies (13, 18, and 21) were excluded.

**Outcome Measures:**

Utilization and yield of genetic testing, and diagnostic rate were assessed as outcome measures and compared to a baseline time period and a genetic testing protocol time period.

**Results:**

There were 201 infants with CHD evaluated by the cardiogenetics program over 3 years. A total of patients 46 patients of the 196 who underwent genetic testing had multiple tests completed. This is a significant decrease from the baseline (247/329, *P* < .0001) and from the genetic testing protocol (29/81, *P* < .0387) time periods. The diagnostic rate was 33% which is significantly increased compared to the baseline rate of 15% (80/524, *P* < .0001) and trends toward a significant increase during the testing protocol rate (25/113, *P *= .0520). The number of dual diagnosis increased to 9 of 201 compared to the baseline (2/524) and the genetic testing protocol (1/113) time periods. The rate of incidental diagnoses altering care increased to 6 of 201 from the baseline (1/524) and the genetic testing protocol (1/113) time periods.

**Conclusion:**

An inpatient cardiogenetics program significantly increases the diagnostic rate, the detection of complex phenotypes with dual diagnoses, the identification of incidental genetic diagnoses associated with changes in care, and significantly decreases the likelihood of multiple tests being completed on an individual patient. Increased medical geneticist involvement in programs that care for infants with CHD should be encouraged to improve patient care and genetic testing utilization.

## 1 INTRODUCTION

Congenital heart disease (CHD) is the most common birth defect and a significant cause of neonatal morbidity and mortality.^1^ Survival rates for even the most severe forms of critical CHD have improved significantly in the modern era, shifting research into focusing on the ability to improve or modify the morbidities associated with surviving critical CHD.^2^ One of the many considerations for a patient with CHD is the etiology of their malformation and genetic assessment of CHD is becoming more emphasized in the care of these patients.^3^, ^4^ Participation of a medical geneticist in care for individuals with CHD has been repeatedly recommended and shown to be beneficial for patients with CHD of all ages in both inpatient and outpatient settings for improvement of patient care and improved resource utilization.^5^, ^6^, ^7^, ^8^, ^9^ It is well established that the presence of a genetic syndrome greatly affects outcomes and that genetic syndromes are often diagnosed later during follow up.^8^, ^10^, ^11^ Studies specific to medical geneticist participation in the care of infants in the cardiac critical care unit are limited, but establish an anticipated diagnostic rate of 25% across all patients.^9^ Our institution aimed to improve care for infants with critical CHD by implementing a cardiovascular genetics or “cardiogenetics” program which increased involvement of medical genetics in the neonatal period to identify genetic conditions more consistently. This article reviews the data from the first 3 years of this program and by comparison to previously assessed baseline data quantifies the impact of the program on patients with CHD.

## 2 METHODS

This project was reviewed by the Children's Hospital of Wisconsin institutional review board and deemed exempt as this information is primarily utilized for internal program quality improvement. The data from this project spans from the start of the cardiovascular genetics program on the first of July 2015 to the end of June 2018.

### 2.1 Program structure

The structure of the inpatient cardiogenetics program has evolved over time. At its initiation in July 2015, the program was based on request for consult by members of the primary team for evaluation by a single medical geneticist (GCG) to evaluate infants with CHD. Patients with isolated atrial septal defects requiring surgical intervention before a year of age did not undergo routine assessment. Due to existing resources specific to patients with trisomy 21, they were excluded from the program in the absence of a specific request for program involvement. In August 2015, it was determined that the service would be more helpful if the medical geneticist was present during the weekly sign out of patients in the cardiac intensive care unit and on the cardiology service as well as the weekly review of upcoming surgical patients. Since that time the medical geneticist (GCG) has regularly attended these meetings. In January 2016, after review for the first 6 months of program data, it was established practice within the Herma Heart Institute that all new patients with CHD admitted to the hospital would be eligible for consultation by the cardiogenetics team. In April 2017, a single genetic counselor (ES) began to support result tracking, result disclosure, and parental counseling. All patients in the program underwent full medical genetics assessment by a single geneticist (GCG). As this data are part of an IRB exempt quality improvement project, we are unable to get the specific data on all patients to determine exactly what percentage of eligible patients underwent assessment. To estimate what number of target patients underwent consults, we took total volume of CHD surgeries in patients less than a year of age and reduced them by 12% to account for our longstanding rate of trisomy 21 in this population (109/891) that is previously published.^12^ However, this number included surgical interventions for atrial septal defects before a year of age and multiple procedures performed on the same patient and is thus an overestimate of how many patients would have been eligible for cardiogenetics consultation.

### 2.2 Inclusion criteria

There were a total of 281 cardiovascular genetics consults over the first 3 years. Of these, there were 207 consults in patients with CHD less than a year of age. After excluding patients with major trisomies (13(0), 18(3), and 21(3)), there were 201 infants with CHD remaining in this cohort. Patients with trisomy 21 were excluded due to existing resources to support patients with this diagnosis. Patients with trisomies 13 and 18 were excluded as cardiac intervention in these patients is still controversial. Data are prospectively tracked and retrospectively analyzed as part of quality improvement for the cardiogenetics program.

### 2.3 Testing and diagnostic rate

All known genetic testing, including prenatal testing and testing completed at outside institutions, is included in our totals. All known genetic diagnoses were recorded. Genetic diagnoses not related to the patients’ cardiac phenotype were recorded as incidental and did not count toward the diagnostic rate. Copy number variants reported as variants of uncertain significance were not included in the diagnostic rate but were included in the abnormal microarray result rate. Cardiac phenotype was recorded by National Birth Defects Prevention Study's classification criteria into their eight “level three” categories: anomalous pulmonary venous return, atrioventricular septal defects, complex, conotruncal, heterotaxy, left ventricular outflow tract obstructions, right ventricular outflow tract obstructions, and septal.^13^ Lesions excluded by this system are classified as other. Comparison data for genetic testing patterns and diagnostic rate are based on data from a previously published cohort from our institution.^12^ Data on two time periods were taken from this cohort, which was matched to the cardiogenetic program population to only include infants without major trisomies. The first time period is “Baseline” which refers to data from January 2010 to December 2013 prior to any interventions where patients may or may not have had evaluation by a geneticist and may or may not have had genetic testing. The second is “Testing Protocol” which refers to data from July 2014 to June 2015, or the year immediately preceding the program where a microarray‐based genetic testing protocol was in place for infants with CHD, but assessment by a medical geneticist was not routine. Further details regarding these cohorts have been previously published.^12^

### 2.4 Data analysis

Descriptive statistics were utilized for cohort characteristics, the proportion of specific cardiac lesions represented within the sample, the frequency of abnormal genetic test results in proportion to tests completed, and presence of genetic diagnosis. As appropriate, Fisher's exact test analyses compared the frequency of abnormal genetic test results between cohorts. A significance level of *P* < .05 was used to evaluate all comparisons.

## 3 RESULTS

### 3.1 Cohort characteristics

Fifty‐six percent of the cohort were male. Seventy‐four percent of the cohort were diagnosed with congenital heart disease prenatally. A summary of all cohorts used in this study (Baseline, Testing Protocol, and Cardiogenetics Program) is illustrated in Table 1. The most common type of lesion in our cohort was conotruncal (*n* = 70) followed by left ventricular outflow tract obstructive lesions (*n* = 63). A breakdown of lesions observed in this cohort are demonstrated in Table 2.

**Table 1:** Cohort characteristics

	Baseline	Testing protocol	Program
n	524	113	201
Male	312	68	112
Diagnosis	80 (15%)	25 (22%)	66 (33%)
Dual diagnosis	2	1	9
Incidental diagnosis	1	1	6
Total tested	329 (63%)	81 (72%)	196 (98%)
Multiple tests	247 (75%)	29 (35%)	46 (23%)

This table demonstrates the rate of diagnosis and testing in the comparison cohorts. The baseline cohort is without any intervention to promote genetic testing or diagnosis in infants with congenital heart disease, the testing protocol cohort is when utilization of a standardized protocol to genetically test infants with congenital heart disease was circulated and promoted, and the cardiogenetics program time period.

**Table 2:** Cardiac lesions seen in cohort and number of patients with diagnosis related to their congenital lesion

Lesion type	Volume	Diagnosis
Anomalous pulmonary venous return	5	3 (60%)
Atrioventricular septal defect	6	4 (67%)
Complex	14	6 (43%)
Conotruncal	70	27 (39%)
Heterotaxy	13	4 (31%)
Left ventricular outflow tract obstruction	63	17 (27%)
Other	2	0
Right ventricular outflow tract obstructions	20	2 (10%)
Septal	8	3 (38%)

This table illustrates the proportion of patients with specific lesions types as defined by the National Birth Defect Prevention Study's criteria and how often they had a diagnosis related to their lesion.^12^

### 3.2 Consult characteristics

**Figure 1:**
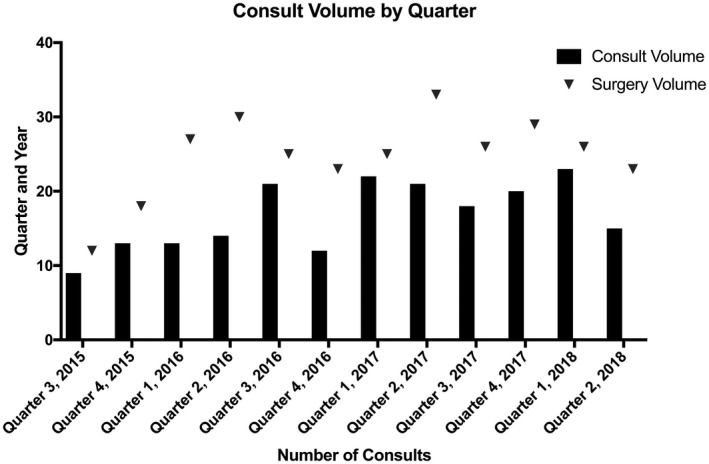
Volume of consults by quarter. Number of consults by quarter with a minimum of 9 and a maximum of 23 with a mean of 17. Number of surgeries completed on infants less than a year of age for congenital heart disease included for reference. The number of surgeries is an overestimate of the number of possible cardiovascular genetics consults due to inability to pull specifics on each patients to exclude those who would have been ineligible for consultation and multiple procedures performed on the same patient. On average 68% of the surgical volume underwent a cardiovascular genetic consultation; however, due to our volume data limitations the number of target patients who underwent consultation is actually higher

The average age at consult was 24 days, with a median of 3 days, and a range of 0‐278 days. The most common consulting service was the neonatal intensive care unit (37%) and cardiology (25%). There was an average of 5‐6 consults per month and consult volume was relatively stable over time as demonstrated in Figure 1.

### 3.3 Genetic testing

The number of genetic tests completed both prenatally and postnatally is summarized in Table 3.

**Table 3:** Genetic testing yield

Test	Baseline	Testing protocol	Program prenatal completed	Program prenatal abnormal	Program postnatal completed	Program postnatal abnormal	Overall program abnormal
Karyotype	15/241 (6%)	6/19 (32%)	14	3	14	2	18% (5/28)
22q11.2 deletion testing	18/215 (8%)	3/18 (17%)	1	0	26	9	33% (9/27)
Microarray	53/222 (24%)	15/76 (20%)	20	9	170	38	25% (47/190)
Exome sequencing	NA	NA	0	0	16	11	69% (11/16)
Other sequencing testing	NA	NA	0	0	40	9	23% (9/40)

This table illustrates the testing pattern and yield for patients split across which tests were completed prenatally and which tests were completed postnatally. This table also gives the total yield of the testing modality across both prenatal and postnatal samples.

#### 3.3.1 Prenatal testing

Twenty‐two (15%) of the 150 patients with a prenatal diagnosis of CHD underwent prenatal genetic testing. Two patients underwent prenatal karyotype alone, eight patients underwent prenatal microarray alone, eleven patients underwent prenatal karyotype and microarray, and one patient underwent prenatal karyotype, 22q11.2 deletion testing, and microarray.

#### 3.3.2 Karyotype

Half of the 28 karyotypes were completed prenatally. Of the remaining 14 postnatal karyotypes, 7 were sent by outside providers prior to genetics involvement in the case. Of the seven karyotypes recommended based on the genetics evaluation, three were sent based on clinical characteristics of the infant and four were sent due to a family history of multiple miscarriages. Both abnormal postnatal karyotypes were karyotypes recommended based on the genetics evaluation.

#### 3.3.3 Microarray

Microarray alone was the most common testing strategy, completed in 144 patients. Chromosomal microarray had a yield of 25%. Six variants of uncertain significance were identified which are not included in the diagnostic rate.

#### 3.3.4 Exome sequencing

Exome sequencing was only considered abnormal if it was conclusively diagnostic. Sixteen patients had exome sequencing completed. The yield was extremely high in this population, with 11 (69%) of these tests being conclusively diagnostic. This likely reflects selection bias and careful resource curation by the medical geneticist.

#### 3.3.5 Other genetic testing

Forty patients underwent molecular testing outside of microarray or exome sequencing. The most commonly ordered test was a heterotaxy panel, which accounted for half (*n* = 20) of the additional molecular tests. Nine (23%) of these tests were conclusively diagnostic. Other molecular testing that was ultimately conclusively diagnostic included testing for common diagnoses seen in this population like CHARGE syndrome, Rubinstein Taybi syndrome, and Noonan syndrome.

#### 3.3.6 Multiple genetic tests

One hundred ninety‐six (98%) patients underwent testing with one or more of the following tests: karyotype, 22q11.2 deletion testing, and/or microarray. Of these 196 patients, 46 (23%) underwent multiple tests. All 22 patients who underwent prenatal genetic testing had more than one test completed and accounted for 48% of the multiple genetic test patients. Even including the prenatal testing patients, this reflects a significant decrease in patients undergoing multiple tests from baseline (247/329, *P* < .0001) and from the testing protocol (29/81, *P* < .0387) periods.

### 3.4 Diagnostic rate

Sixty‐six of 201 (33%) patients had a genetic diagnosis related to their congenital heart disease. The most common diagnosis was 22q11.2 deletion syndrome in 15 patients (7%). This is a significant increase in diagnostic rate from baseline (80/524, *P* < .0001) time period and trends toward a significant increase from the testing protocol (25/113, *P* = .0520) time period. Table 2 demonstrates the spread of diagnoses by cardiac lesion type. It is interesting to note the low number of diagnoses in the RVOTO compared to other groups with a substantial number of patients.

#### 3.4.1 Dual diagnoses

Nine patients had dual diagnoses, reflecting 14% of all patients with a genetic diagnosis related to their CHD. Four of these patients had more than one distinct chromosomal anomaly not associated with a translocation. Four patients had combinations of two single gene disorders or a single gene disorder and a chromosomal anomaly. One patient had a diagnosis of 22q11.2 deletion syndrome in addition to a symptomatic congenital TORCH infection. This is a significant increase from baseline (2/524, *P* = .0003) time period. While it illustrates a clear, clinically relevant increase from the testing protocol (1/113) time as well, this did not reach statistical significance due to a low number of total patients in that time period. The dual diagnoses made prior to the cardiogenetics program were two patients with more than one distinct chromosomal anomaly found on microarray and one patient with 22q11.2 deletion syndrome who was found to also have Cornelia de Lange syndrome on exome sequencing performed by the genetics service at a few years of age.

#### 3.4.2 Incidental diagnoses

There were six incidental genetic diagnoses made as a result of medical geneticist consultation that did not explain the patient's congenital heart disease, but directly altered care. Two of these were predispositions for pediatric cancers (Wilms Tumor and Hepatoblastoma). The remaining four diagnoses were congenital hearing loss, dystrophic epidermolysis bullosa, Marfan syndrome, and sex reversal due to SRY translocation. This is a significant increase from the baseline (1/524, *P* = .0023) time period. While a clear, clinically relevant increase from the testing protocol (1/113) time as well, this did not reach statistical significance due to a low number of total patients in this time period. The incidental diagnoses made prior to the cardiogenetics program were sickle cell anemia and hemophilia.

## 4 DISCUSSION

Our data demonstrates that increasing medical geneticist participation in the care of infants with critical CHD reduces the number of patients undergoing multiple genetic tests, increases the diagnostic rate, increases the detection of complex phenotypes and dual diagnoses, and increases the number of incidental genetic diagnoses associated with changes in care.

The most important finding on review of the program data is that there is an increase in dual diagnoses and incidental diagnoses. While these were present in the baseline and testing protocol cohorts, four of five patients that fit into these categories prior to the program had diagnoses that were made without evaluation. For example, the patient with sickle cell anemia was diagnosed by newborn screening, the patient with hemophilia was diagnosed based on known family history with a family that actively communicated this risk from the prenatal period on, and two of the dual diagnosis patients were multiple chromosomal anomalies made by microarray testing. The patient with a complex dual diagnosis of 22q11.2 deletion syndrome and Cornelia de Lange syndrome was made after being seen by the genetics service at a few years of age for being more severe than expected for the diagnosis of 22q11.2 deletion syndrome prompting exome sequencing to be completed. For patients in the cardiogenetics program, dual diagnoses and incidental diagnoses were made within the first few months of life which allowed for earlier appreciation of patient risk and changes to care as needed prior to complications presenting. Given the increasing evidence that outcomes for patients with CHD are strongly influenced by genetic factors, early and complete identification of these factors has significant potential to help patients.^11^ This data are compelling that medical geneticist evaluation provides significant benefit to infants with CHD. It also should be noted that despite the proven utility of these consultations, unfortunately access to medical geneticists is limited.^3^

Another interesting finding in our data are that only 15% of our patients who had a prenatal diagnosis of CHD undergo prenatal genetic testing. It is unclear as to why this is, anecdotally it seems that families perceive prenatal genetic testing to be only for the purposes of decision making regarding termination vs medical care for the infant. Thus, prenatal genetic testing may be an area for enhanced education to allow for earlier diagnosis and more timely precision care in patients diagnosed with critical CHD prenatally.

Limitations of this data include the potential for selection bias of who undergoes cardiogenetic assessment resulting in an enrichment of patients with genetic diagnoses, more accessibility to genetic testing over time, inability to concretely demonstrate the number of target patients who underwent cardiogenetics assessment due to being an IRB exempt quality improvement project without access to this data, and inconsistency of the program over time due to evolutions of service. Even with these limitations, this data strongly supports the utility of increased formal, standardized medical geneticist involvement in the care of infants with critical CHD. Based on our program experience we would recommend participation in weekly patient signs outs and weekly surgical planning conferences as this can extend the reach of the medical geneticist across a wider array of patients in a time effective manner. Future areas of evaluation include looking at timing of genetic testing return and diagnosis in comparison to timing of surgery and how the timing of these affect care.

## CONFLICT OF INTERESTS

The authors have no conflicts of interest or disclosures. This work had no direct funding. The clinical program described in this article is supported by Medical College of Wisconsin's Department of Pediatrics.

## AUTHOR CONTRIBUTIONS

GCG: Concept/design, data collection, data analysis/interpretation, drafting article, critical revision of article, approval of article.

ES: Data analysis/interpretation, critical revision of article, approval of article.

MGE: Concept/design, data analysis/interpretation, critical revision of article, approval of article.
